# Neurophysiological and behavioural markers of compassion

**DOI:** 10.1038/s41598-020-63846-3

**Published:** 2020-04-22

**Authors:** Jeffrey J. Kim, Stacey L. Parker, James R. Doty, Ross Cunnington, Paul Gilbert, James N. Kirby

**Affiliations:** 1 School of Psychology, The University of Queensland, Brisbane, Queensland Australia; 2 Center for Compassion and Altruism Research and Education, Stanford University School of Medicine, 2490 Hospital Drive, Suite 106, Stanford, California USA; 3 School of Allied Health and Social Care, University of Derby, Derby, DE22 1GB United Kingdom

**Keywords:** Neuroscience, Psychology

## Abstract

The scientific study of compassion is burgeoning, however the putative neurophysiological markers of programs which actively train distress tolerance, such as Compassionate Mind Training (CMT), are less well known. Herein we offer an integrative, multi-method approach which investigated CMT at neural, physiological, self-report, and behavioural levels. Specifically, this study first assessed participants’ neural responses when confronted with disappointments (e.g., rejection, failure) using two fundamental self-regulatory styles, self-criticism and self-reassurance. Second, participant’s heart-rate variability (HRV) – a marker of parasympathetic nervous system response – was assessed during compassion training, pre- and post- a two-week self-directed engagement period. We identified neural networks associated with threat are reduced when practicing compassion, and heightened when being self-critical. In addition, cultivating compassion was associated with increased parasympathetic response as measured by an increase in HRV, versus the resting-state. Critically, cultivating compassion was able to shift a subset of clinically-at risk participants to one of increased parasympathetic response. Further, those who began the trial with lower resting HRV also engaged more in the intervention, possibly as they derived more benefits, both self-report and physiologically, from engagement in compassion.

## Introduction

Access to mental health treatments have improved drastically over the last two decades, and yet rates of mental health problems have not decreased, with some research indicating they are on the rise^1–3^. How does one make sense of this apparent paradox? Competing explanations suggest population growth, lack of treatment engagement, aging, and the possibility that although 12-month prevalence rates might not have decreased, length of mental health episodes have^1,4^. We offer an alternate possibility – namely the role of pathogenic self-criticism within modern society – which has increased the number of individuals with mental health issues, and also blunted the impact of available treatments on mental health problems. Importantly, the clinical literature is clear - there is substantial evidence that self-criticism contributes to mental health problems and impedes recovery^5,6^.

In a recent major review of 48 studies, self-criticism is associated with: eating disorders, depressive disorders, social anxiety disorder, personality disorder, psychotic symptoms and interpersonal difficulties^6^. It is also associated with self-harm and suicide risk^6^. Self-criticism interferes with goal pursuit^7^, and is associated with problematic perfectionism, which has been increasing steadily over the last 20 years^8^. Ever since Freud argued that depression arose from ‘anger turned inward’^9^, i.e., a form of self-criticism, it has been a therapeutic target for many schools of therapy^10^. Furthermore, Whelton and Greenberg^11^ showed that it was when self-criticism generated hostile emotion to the self, that individuals felt particularly overwhelmed and defeated by their self-criticism. Hence, it’s not just the content of a self-criticism that can be pathogenic, but the emotional intensity in which the criticism is delivered and experienced by the self that is important.

Exploring the forms and functions of self-criticism, Gilbert and colleagues^12^ found two separate forms and functions relating to criticism. One form concerns a sense of inadequacy where the function is to improve oneself and avoid making mistakes, and the other form is a self-hating where the function is to punish or remove undesired aspects of the self. Furthermore, a study of 279 outpatients with mental health problems^13^ confirmed the importance of distinguishing self-hatred from self-improvement forms of self-criticism. These researchers also identified that self-hatred was a key mediator of the relationship between shame and poor mental-health outcomes^13^. While the forms and functions of self-criticism can be explored with self-report and clinical observation, research on the neurophysiological processes by which self-criticism exerts its pathogenic effects have also been explored. In an fMRI study, Doerig *et al*.^14^ invited participants to focus on self-critical versus neutral adjectives. They found activation of dorsomedial and lateral frontal pathways, as well as the amygdala and posterior cingulate, when engaged in self-criticism. Longe *et al*.^15^ used a different methodology and presented participants with written stimuli which describe a mistake, setback, or failure, and manipulated the emotionality of the vignettes (i.e., negative versus neutral) as well as the perspective to adopt (i.e., self-criticism verses self-reassurance). These factors were associated with unique neural substrates, whereby self-criticism was associated with activation within the DLPFC, and self-reassurance the anterior insula^15^.

Whilst important research into the neural and self-report bases of criticism have been conducted, however, research has yet to continue an understanding of factors which may dampen criticism’s pathogenic effects, such as self-compassion and/or self-reassurance. Self-reassurance is the ability to be encouraging, resilient, and aware of one’s positive qualities and capabilities in the face of setbacks, mistakes or failures^16^. Importantly, the ability for self-reassurance has been shown to protect against symptoms of psychopathology, such as depression and anxiety^16^, whereas self-criticism has been shown to predispose toward their development^17^. Within a Compassionate Mind Training (CMT) framework, self-reassurance is the cognitive relating style used when adopting a compassionate-mind^16^. Compassion is defined as, “a sensitivity to suffering in self and others, with a commitment to try and alleviate and prevent it (p. 2)^18^”. Thus, if the motive of a behavior or cognitive relating style is to reduce suffering there are many ways one can do this, such as being kind to oneself, as well as being self-reassuring^16,18^.

Within the present research, we will replicate and extend Longe and colleague’s fMRI paradigm^15^ by incorporating a focal emotion contrast (i.e., negative minus neutral), to further examine how self-criticism verses self-reassurance might modulate neural responses to negative emotion. In addition, our integrative and multi-method approach, which first assessed participants’ neural markers of self-critical versus self-reassuring motivations with fMRI, also tracked the same participants through a compassionate mind training protocol with markers of physiology, specifically heart-rate variability (HRV), assessed pre- and post- a two-week self-directed engagement period.

Extensive neuroimaging research has associated activation of a core salience network (SN), involved in the processing of affective, painful stimuli^19–21^. Comprised of two key regions, the anterior insula and anterior cingulate, activation of this network has been implicated across pre-existing compassion and empathy fMRI studies to date^22^. Importantly, whilst affective stimuli have been shown to produce activation in this network, cultivating compassion has been shown to reduce the salience and experience of negative emotions^23^. Therefore, we expect to observe activation of this network for emotional written vignettes and anticipate that self-criticism would boost this activation whereas self-reassurance would suppress this activation. An additional network reputed to be involved in compassion-related processes is the default-mode network (DMN)^24,25^, jointly involved in self-referential and mentalizing processes^25,26^. Critically, recent efforts have sought to understand the role of default-mode function in clinical interventions^27^, specifically with forms of mental training, such as meditation, which have been associated with alterations in DMN activity and functional connectivity^28^. Therefore, we anticipate potential recruitment of this network in parallel with SN activation when engaged in self-criticism and self-reassurance motivations, given our task is self-referential and mentalizing in nature.

Next, in our brief Compassionate Mind Training (CMT) intervention, we utilized the practice “Cultivating the Compassionate Self”, which is a central compassionate mind training practice within Compassion Focused Therapy (CFT). This intervention comprises four core elements; (1) grounding and body posture, (2) soothing-rhythm breathing, (3) mindfully noting when self-criticism arises, and switching to (4) cultivating the compassionate-self^29^. With this training protocol, we assessed participant’s HRV at both rest and during the compassion exercise. Higher HRV is indicative of higher parasympathetic nervous system outflow via the Vagus nerve^30–33^, activity which have been associated with increased states of contentment, calmness, safeness^31^. Notably, when the sympathetic nervous system is recruited for defensive or other behavioural activation systems, HRV is reduced^31^. Previous research has identified how these CMT exercises can significantly increase baseline HRV and reduce symptoms of depression, anxiety, stress, and fears of self-compassion^34,35^. Hence, we predict our compassion intervention will, compared to baseline, increase HRV. As an additional exploratory analysis, we will examine the percentage improvement of participants who move out of a clinical ‘at risk’ range of HRV when engaged in compassion, as identified in a recent paper which assigned elevated cardiac risk to low resting HRV^36^. We will also assess the degree to which two-weeks of training is sufficient to reduce fears of self-compassion, and we will explore the relationship between HRV and behavioural markers of engagement in compassion training across the two-week self-directed engagement period, as assessed via frequency of accessing the training link.

## Results

### Neural activity for imagery toward emotional stimuli

**Figure 1:**
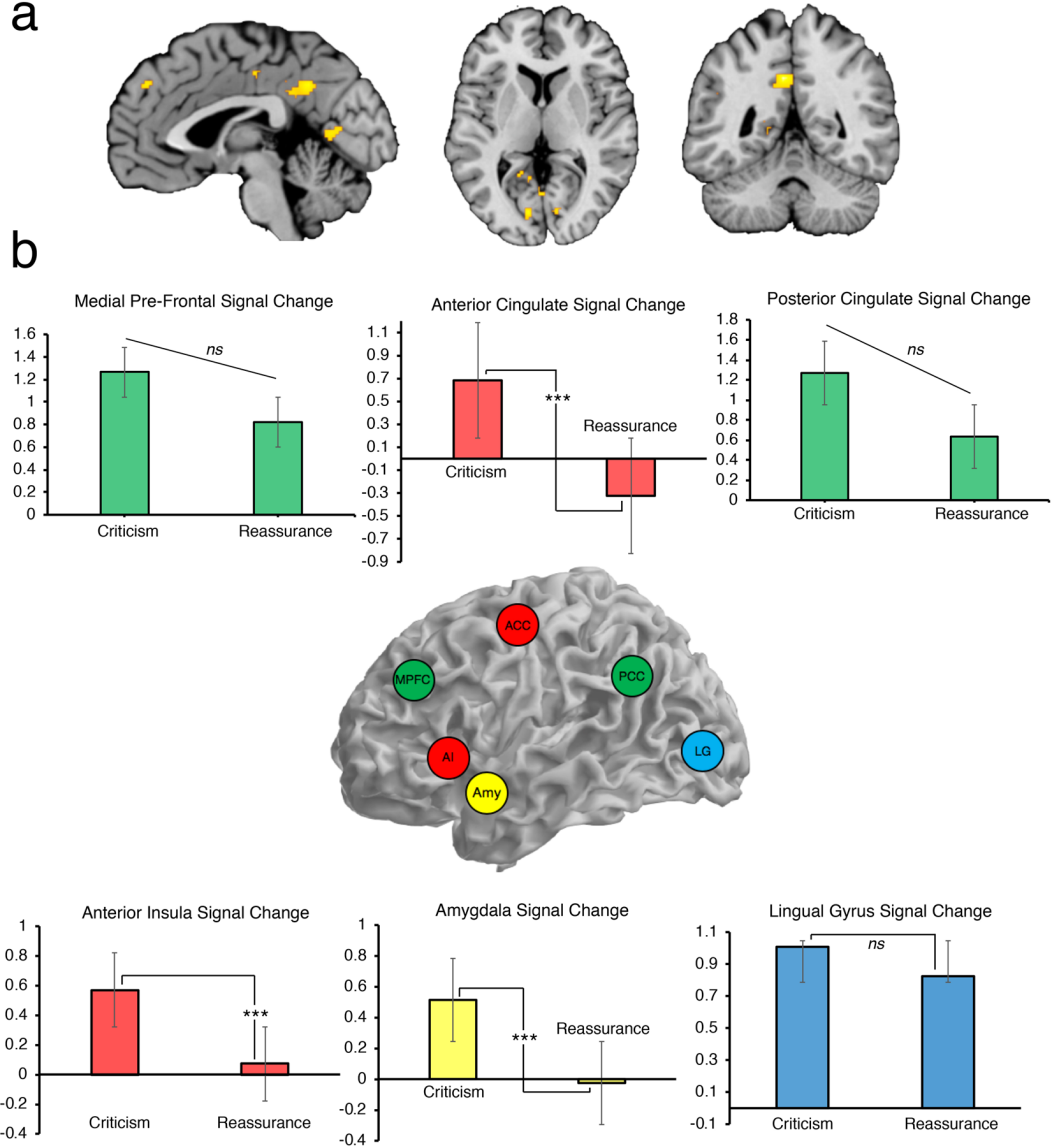
Thresholded whole-brain activation for the Emotional – Neutral contrast, revealing an overall effect of emotional imagery, and ROI approach comparing criticism to reassurance. (**a)** Left: Sagittal image of Posterior Cingulate, Lingual Gyrus, and Medial Frontal Cortex (x = −2). Middle: Axial image of Lingual and Calcarine Gyri (z = 4). Right: Coronal image of Posterior Cingulate (y = −50). Activation corrected at *p*FWE < 0.05, and coordinates reported in MNI-space. (**b**) ROI approach comparing self-critical imagery to self-reassuring imagery, with % signal change on the Y-axis. Top: Comparison of MPFC (2 46 36), ACC (0 14 36), and PCC (4 52 36) % signal change. Middle: Inset which depicts rough spatial location of ROIs. Bottom: Comparison of Left AI (−26 10 −14), Left Amygdala (−28 −4 −12), and Middle Lingual Gyrus (0 −68 6) % signal change. Coordinates reported in MNI-space. Error bars indicate standard error. N = 40. ***p < 0.001, *ns p* > 0.05.

To test whether emotionally salient stimuli (written vignettes) elicited activation in brain regions which code for negative affect, we examined brain activity during imagery toward emotional minus neutral statements. Within-subjects contrasts revealed significantly greater activation for emotional compared with neutral stimuli, tested across the whole-brain, found in regions of the SN and DMN, as well as regions of the occipital cortex (see Fig. 1a, all p*FWE’s* < 0.05). This neural activation included: left PCC, left calcarine gyrus, left and right lingual gyrus, left MPFC, and left ACC (See Supplementary Table 1 for peak co-ordinates and statistical values).

### Neural activity for imagery during self-criticism and self-reassurance

Next, we conducted an ROI approach to examine differences in neural activation when participants engaged in self-criticism and self-reassurance. We extracted neural responses from the ACC, MPFC, PCC and the lingual gyrus, to extend upon significant whole-brain activation as identified above. We also extracted an ROI from the left amygdala and left insula cortex, as previous research across multiple studies have linked activity in these regions with compassion-related processes^22,37,38^. Voxel-wise coordinates for each ROI were defined as each participant’s peak cluster identified with the AAL labelling tool under the WFU-pick atlas in SPM12, from the emotional-neutral contrast, for both self-criticism and self-reassurance. As can be seen in Fig. 1b, significantly greater neural activation within the AI, ACC, and the amygdala, was identified for self-criticism as compared with self-reassurance (paired-sample t-tests, all *p’s* < 0.001). Activity within the MPFC, PCC, and lingual gyrus did not differ between conditions (Fig. 1b, paired sample t-tests, *p* > 0.05, *ns*).

### Physiology at rest versus intervention

**Figure 2:**
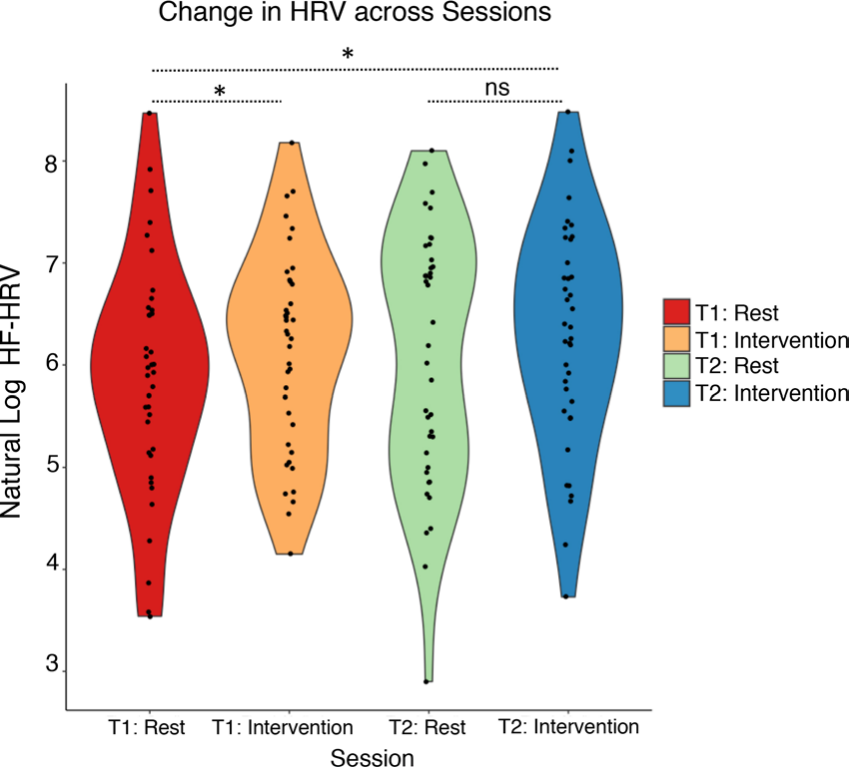
Overall changes in physiology (HRV) derived from our compassion intervention. Changes in HRV, expressed as natural log units, are depicted at rest and during intervention, at both pre- and post- two-week intervention. N = 38, accounting for listwise deletion. *p < 0.05, *ns p* > 0.05.

To derive a measure of parasympathetic response with our HRV, we created natural-logarithm units of high-frequency components (0.15–0.40 Hz) of participants ECG-trace, for each 15-minute rest and 15-minute intervention component, at both pre- and post- two-week training. As can be seen in Fig. 2, a two-way ANOVA revealed a significant within-subjects effect of session (i.e., rest vs intervention), whereby HRV was higher when engaged in the compassion intervention than at rest (*F*(1,36) = 5.673, *p* < 0.023). However, we observed a non-significant within subjects effect of order (i.e., pre and post two-week training), *F*(1,36) = 2.694, *p* = 0.109), whereby rest versus intervention did not differ between timepoints, and a non-significant interaction (*F*(1,36) = 0.033, *p* = 0.857. Follow-up t-tests revealed significant increases in HRV from T1 rest to T1 intervention (t(37) = 2.310, *p* < 0.027), as well as from T1 rest to T2 intervention (t(36) = 2.667, *p* < 0.011). T2 rest to T2 intervention was non-significant (t(38) = 1.813, *p* = 0.078).

### HRV-shift for clinically at-risk participants

**Figure 3:**
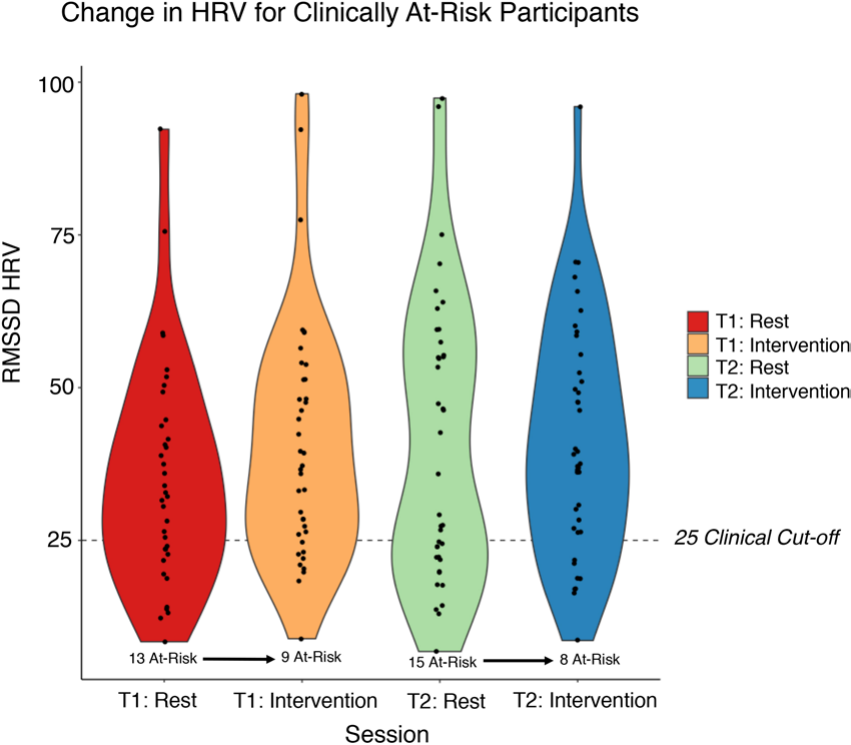
Percentage improvement of a subset of participants shifted out of a clinically at-risk range of poor resting-HRV, after engagement in compassion. X-axis depicts pre- and post- two-week training, at either rest or intervention (compassion meditation), and Y-axis depicts RMSSD HRV value. Dashed-line depicts clinical cut-off (25), and subplot text describes the number of clinically at-risk participants which moved above the clinical cut-off after engagement in compassion. N = 38, accounting for listwise deletion.

Next, we assessed the percentage improvement of participants who were able to shift out of a clinically at-risk range of low resting-HRV, as defined empirically^36^. In accordance with these guidelines, we report raw RMSSD HRV scores for interpretation, and note RMSSD HRV is considered a reliable and valid time-series metric of vagally-mediated HRV^33,39^. As can be seen in Fig. 3, four and seven clinically-at risk participants were shifted above the clinical cut-off of 25 RMSSD^36^ when engaged in the compassion intervention versus at rest, at either pre- and post- two-week training, respectively.

### Relationship between physiology and behaviour

**Figure 4:**
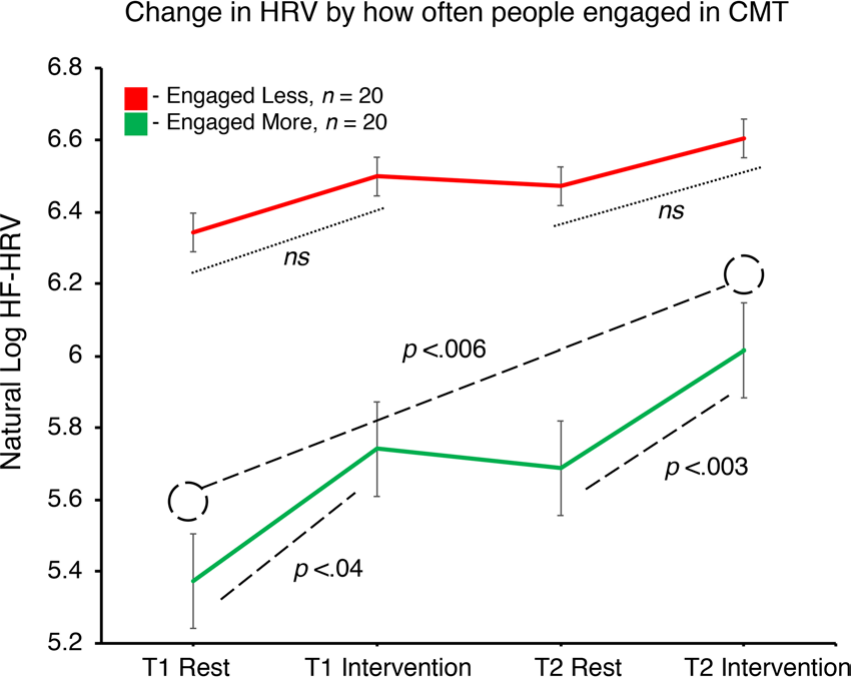
Relationship of HRV and Behaviour (frequency of engagement in compassion), whereby data were median split into those with Less versus More engagement of the compassion recording across the two-week engagement period. N = 38, accounting for listwise deletion. Error bars indicate standard error. **p < 0.01, *p < 0.05, *ns p* > 0.05.

Next, we examined the relationship between HRV and how often participants engaged in compassion training across the two-weeks. From behavioural metrics of how often participants accessed the compassion exercise across the two-week period, which we assessed as frequency of recording access from the experiment website, we median split the data into those who engaged More versus Less. Those who engaged Less (N = 20) accessed the recording on average 3 times across the two-week period *(M* = 2.9, *SD* = 1.2, range = 1–4), in contrast, those how engaged More (N = 20) accessed the recording on average 9 times across the two-week period (*M* = 9.1, *SD* = 3.1, range = 5–15). A 3-way mixed ANOVA revealed a significant between subjects effect, whereby those who engaged Less had higher HRV overall, *F*(1,35) = 6.746, *p* < 0.014 (Fig. 4). We observed a non-significant effect of session order, whereby HRV pre- versus post- were not significantly different (*p* > 0.05, *ns*). The three-way interaction term between participant engagement, session type, and session time was non-significant (*p* > 0.05, *ns*). Follow-up paired t-tests revealed significant increases in HRV for those who engaged More across the two-weeks, from rest to intervention at Time 1 (*p* < 0.04), rest to intervention at Time 2 (*p* < 0.003), and from Time 1 rest to Time 2 intervention (*p* < 0.006). These effects were non-significant for those who engaged Less (*p’s* > 0.05, *ns*).

### Pre-post self-report differences

Finally, we observed a significant decrease in fears of expressing compassion to the self^40^. Paired sample t-test (Time 1: M = 54.53, SD = 22.83, Time 2: M = 33.68, SD = 15.69), t(39) = 5.683, p < 0.001.

## Discussion

This study conducted an integrative, multi-method approach which first investigated two fundamental self-regulatory styles (self-criticism and self-reassurance) with fMRI, and second, measured participant’s HRV, a marker of parasympathetic response, during compassion training, pre- and post- a two-week self-directed training period.

Upon investigation of neural correlates of self-critical versus self-reassuring motivations, our manipulation check for a focal emotion contrast identified increased neural activation across SN and DMN, as well as foci within the visual cortex (Fig. 1a). Importantly, a ROI approach revealed self-reassurance and self-criticism modulated neural responses to negative emotion, in regions such as the AI, ACC, and amygdala (Fig. 1b), whereby self-reassurance was associated with a decrease in neural reactivity within these regions, as opposed to self-criticism, which boosted neural activation within these regions. Interestingly, activity in MPFC, PCC, and lingual gyrus (within visual cortex) did not differ between self-criticism and self-reassurance.

Overall, these findings replicate previous research which identified SN activity as associated with compassion-related processes^19,22^, and we are the first to explicitly afford the DMN a putative role for compassion-related processes, both self-critical and self-reassurance motivations. Interestingly, in direct contrast with the results of Longe *et al*.^15^, our data show that both criticism and reassurance modulate similar neural pathways, rather than operating on distinct neural regions (i.e., DLPFC) or networks (i.e., SN). However, we note the focus of the current experiment were to identify neurophysiological correlates of compassion and criticism, as well as physiological shift during compassion cultivation. More work is needed to directly test for similarities and/or differences in these neural systems, as this was not the aim of the current experiment.

Second, with our CMT protocol, we identified increased HRV during compassion training versus at rest, indicative of increased parasympathetic response (Fig. 2). Overall these findings replicate previous research which has identified increased HRV during meditation practices^34,35,41^, such as compassion and mindfulness training. In addition, our brief compassionate mind training protocol was also able to shift a subset of participants out of a clinically at-risk range of 25 RMSSD HRV at rest (Fig. 3).

Third, we identified resting HRV predicted behavioral markers of engagement in compassion training across the two-weeks (i.e., levels of recordings accessed). Specifically, individuals who had lower resting HRV engaged more in the intervention and derived more physiological benefit (i.e., increases) in HRV from rest to intervention, at both pre- and post- two-week intervention (Fig. 4). In addition, we note also that individuals with higher resting HRV engaged less in compassion during the two-week training period, and did not derive significant physiological shift from rest to intervention across pre- and post-training (Fig. 4). Last, our data also identified a significant reduction in fears of self-compassion after the two-week intervention, which provides a validation of the effects of CMT at the self-report level.

Importantly, our HRV data suggests individual differences in compassionate responding exist, whereby for some individuals significant HRV-change across sessions did not occur (Fig. 4). Participants never received feedback on their HRV scores or what this data may indicate, yet those with lower resting HRV were also those which engaged in the practice more frequently and benefitted physiologically as a result. Thus, this finding can potentially position our results in the context that individuals whom really needed the practice, physiologically, did use it. These findings may extend Gilbert’s evolutionary and neurophysiologically-informed model of compassion^29^, to highlight how individual differences across threat- versus self-soothing systems may relate to HRV-response and engagement. For example, CMT was designed as an adjunct to traditional psychotherapies for those whom are treatment non-responsive^34^. The clinical implications of these data are fascinating - given our data show increases in HRV during a compassion meditation state, to what extent are these differences not apparent for those who might have fears, blocks or resistances to compassion^40^? Furthermore, how intensive might training need to be in order to provide benefit to these individuals?

In accordance with our data that compassion and criticism may recruit similar neural networks and regions, we suggest that the act of imagery generated while engaging in self-criticism may act upon similar subcortical routes for processing threatening stimuli in general, processes which may be stimulated automatically and unconsciously^42–44^. Interestingly, previous research has identified subconscious pathways for threat processing in the amygdala and visual cortex (lingual gyrus) apparent even under conditions of cortical blindness^45,46^, therefore we suggest that amygdala and lingual gyrus activation likewise identified within our research may subsume a similar route.

The aim of this integrative project was to isolate specific components of CMT, and to balance a multi-modal design with a brief two-week training component. Therefore, we utilized a within-groups approach as our *a priori* aim was to link neurophysiological, behavioural, and self-report data. However, to extend upon this work with a between groups design would be of benefit, to determine whether CMT is more effective than other mental training, such as mindfulness. We would argue that CMT training for threat would be more robust than mindfulness, given CMT is at its core comprised of training to try and be helpful to observed suffering and/or distress. Whereas mindfulness meditation may instead direct attention toward a mantra and/or alternate locus of attention^47^, and to effectively dissociate from threat, given the nature of its attention training paradigm. We anticipate future work which could investigate physiological (HRV) shift to both safety and threatening scenarios would be a direct test of this hypothesis, with procedures such as the Trier Social Stress Test^48^. Here, we anticipate that CMT would enable participants to better regulate threat and return to parasympathetic response more quickly after threat, as opposed to mindfulness training. Given recent research has identified that acting altruistically in the face of threat can reduce both physical and psychological pain^49^, it is possible that CMT may likewise hold an analgesic or moderating effect on threat and pain.

Furthermore, although the median split approach has been questioned^50^, we affirm the robustness of this technique given the presence of uncorrelated or non-linear related variables^51,52^. Given we now better understand neurophysiological markers of compassion within a healthy control sample, however, we look forward to future research which could potentially consider a control versus a clinically-relevant sample (i.e., depression and anxiety). Our HRV data may tentatively support a distinction between these groups, first given participants’ baseline HRV varies considerably, and second that we were able to shift a subset of at-risk participants above the clinical cut-off when engaged in soothing compassion states (Fig. 3). In addition, whilst we have identified the efficacy of CMT to down-regulate neural markers of threat and increase physiological and self-report makers of safety, soothing, and regulation, we note however that we did not observe a baseline HRV shift for participants at pre- to post-. Therefore, in order to better understand who may benefit from cultivating compassion, more work is needed, potentially with a more longform approach, to assess for a true baseline shift, in both “normal” and clinically relevant samples.

## Methods

### Participants

40 participants (Mean age = 22 years, SD = 0.49, 27 female) took part in the present study. The University of Queensland Health and Behavioural Sciences, Low & Negligible Risk Ethics Sub-Committee approved the experimental protocol, and this project complies with the provisions contained in the *National Statement of Ethical Conduct in Human Research* and complies with the regulations governing experimentation on humans. Participation was voluntary and anonymous, and subjects provided informed, written and/or electronic consent. Experimental stimuli and procedures, analysis code, data, and statistical output are available in an OSF repository (10.17605/OSF.IO/6H9MD), and fMRI data are available on a University of Queensland Research Data Manager (RDM) server, with access available upon request.

### fMRI Stimuli

We created 60 written stimuli in total, consisting of a personal mistake, setback or failure. 30 statements were of emotional valence whereas 30 were neutral (i.e., “I fail to keep up with my commitments in life”, and “I keep up with my commitments in life”, respectively). Our neutral stimuli were created to describe a non-emotive, non-intense control to counterbalance the emotional stimuli set. For both emotional and neutral sets we assessed two metrics, valence (1–5, where 1 = Very Unpleasant) and intensity (1–5, where 1 = Not Intense). Our emotional statements (*n* = 30) were revealed to be sufficiently unpleasant (*M* = 1.89) and intense (*M* = 3.54), with all neutral statements (*n* = 30) described as less unpleasant (*M* = 3.80) and comparatively not intense (*M* = 2.34).

### fMRI design

Within the scanner pre- two-week training, we examined participant’s neural responses to the validated (affective and neutral) written stimuli when engaged in self-criticism and self-reassurance. After each trial within a block of either self-criticism or self-reassurance, participants rated how intense their degree of self-criticism or self-reassurance was to each statement (button-press on an MR-compatible button box which ranged from 1–4, where 1 = not very intense, and 4, very intense). A typical trial consisted of stimuli presented for a 6 second duration, followed by a rating of intensity for a 3 second duration, and an inter-trial-interval of 0.5 seconds. The first order of instruction for a particular block, that is, self-reassurance verses self-criticism, was counterbalanced for a total of 8 blocks. As our focal contrast, we manipulated the emotionality of the statements within scan runs (“emotive” vs “neutral”), in a counterbalanced order across participants. 30 statements were quasi-randomized across participants and presented for a total of 30 trials per fMRI run (~6.5 min total duration) over a total of 8 repeated fMRI runs. Participants were given 10 practice trials of emotional and neutral stimuli, and rated stimuli on intensity.

### HRV design

Approximately a week after the fMRI experiment, participants listened to a standardized 15-minute recording of the CFT exercise “Cultivating the Compassionate Self”, with HRV collected pre- and post- a two-week self-directed engagement period. The guided exercise was recorded in English by a Clinical Psychologist and expert in CFT. A website to facilitate this project during the two-week engagement period between Time 1 and Time 2 was created to house the audio recording and various instructions/helpful tip sheets for participants.

### fMRI acquisition and pre-processing

We collected our fMRI data on a 3-Tesla Siemens Trio MRI scanner utilizing a 64-channel head-coil. A gradient-echo, echo-planar “fast imaging” (EPI) sequence were used to acquire functional images, with the following sequence parameters: 60 horizontal slices (2 × 2-mm in-plane voxel resolution and 2-mm slice thickness plus 10% gap), repetition time (TR) 1000 ms; echo time (TE) 30 ms. Eight identical fMRI runs of 292 images (6 minutes each) were acquired. A 3D high-resolution, unified and denoised T1-weighted MP2RAGE image across the entire brain was also acquired and used as anatomical reference for subsequent pre-processing in SPM12 (TR = 4000 ms, TE = 2.93 ms, FA = 6°, 176 cube matrix, voxel size = 1-mm). Functional imaging data were pre-processed and analyzed using SPM12, implemented in MATLAB. Structural T1-scans were co-registered to the average of the spatially realigned functional slices. Next, an inbuilt segmentation routine was applied to register each structural T1-image to the standard MNI template in MNI space. These transform parameters elicited from segmentation were subsequently applied to all realigned images, resliced to a 2 × 2 × 2-mm resolution and smoothed with 6-mm full-width-at-half-maximum (FWHM) isotropic Gaussian kernel.

### fMRI first and second-level analyses

For first-level data analysis, block-related neural responses to stimuli were modelled as 2 separate conditions (all combinations of emotional/neutral, self-criticism/self-reassurance) and convolved with the canonical hemodynamic response function (HRF). For group level analysis, wholebrain contrasts of emotional-neutral stimuli overall were reported at a cluster-level threshold of *p* < 0.05, corrected for family-wise error, with clusters formed with a voxel-level height threshold at *p* < 0.001, uncorrected. Brain regions shown to be significant had their anatomical labels identified with the Automated Anatomical Labelling (AAL) toolbox implemented in SPM12^53^. Next, in order to examine correlations between the level of neural activation (i.e. difference in response between emotion verses neutral) and the mindset participants engaged in (i.e. self-criticism versus self-reassurance), we performed additional region of interest (ROI) analyses. For each ROI, we identified peak clusters which showed significantly greater activation overall for emotion vs neutral stimuli, and used these coordinates to extract the average contrast parameter estimates (i.e. levels of activation, Beta weights) with 5-mm radius spheres centered on those peaks for each mindset (i.e., self-criticism and self-reassurance). We then used SPSS to examine the correlation between neural responses and the mindset participants engaged in when processing neural responses to emotional stimuli, and used the R Statistical Programming Language to visualize both regions-of-interest results, as well as subsequent HRV data.

### fMRI trial-by-trial intensity ratings

Analysis of participant’s mean level of intensity for reassurance (emotional statements: *M* = 2.45, *SD* = 0.48, neutral statements: *M* = 2.63, SD = 0.64) and criticism (emotional statements: *M* = 2.92, *SD* = 0.45; neutral statements: *M* = 2.07, *SD* = 0.52) revealed intensity ratings were significantly higher for critical (emotional – neutral) but not for reassuring (emotional – neutral) trials (t(38) = 7.300, *p* < 0.001, and t(38) = −1.372, *p* = 0.178, *ns*, respectively). Inspection of a correlation matrix revealed intensity ratings for reassurance and criticism during emotional trials were correlated (R = 0.47, *p* < 0.003).

### HRV data acquisition, pre-processing and analysis

We collected our heart-rate variability (HRV) data with a portable Schiller Systems Medilog electrocardiogram (ECG) device. These recorders of the “AR12plus” type utilize a 3-lead bipolar ECG amplifier, and we positioned each lead on the chest, to maximize ECG amplitude and minimize muscle artifacts. After data collection, data were digitized, pre-processed, and extracted using Schiller’s proprietary Medilog Darwin software package, which processes the ECG biosignal to comply with accepted guidelines in the field for measurement and analysis of HRV data. Via visual inspection of the ECG biosignal, and after application of an algorithm which automatically filters for normal and non-normal beats, we excluded signal artifacts and irregular (non-normal) beats not removed from the initial application of the pre-processing algorithm. Therefore only the normal beats (via R-wave peak detection) were extracted. The sampling period was 1000 Hz at a time resolution of 250 μs (microseconds), and we utilized linear detrending. After initial pre-processing, high-frequency (HF) HRV components (i.e., 0.15–0.40 Hz) of each participants’ ECG trace were extracted upon application of the discrete Fourier transform (DFT) of the beat-to-beat interval times-series. We also extracted raw RMSSD HRV components, namely the Root Mean Square of Successive Differences of the HRV beat-to-beat timeseries. Data were coded as being from either Time 1 or Time 2, at either Rest or Intervention. Signal averages for each of these periods were created from the bins downloaded from Medilog Darwin (i.e., 4*15-minute bins, across Rest and Intervention, at both Time 1 and Time 2). We did not measure or control for respiration during the ECG recording, yet we did attempt to control for additional sources of variance such as posture, exercise, alcohol, caffeine intake, and time of testing. HRV data were transformed to the natural logarithm (base of the mathematical constant *e*) in order to better approximate normality. Missing data for HF-HRV and RMSSD were less than 0.03% and 0.04%, overall, and missing data were counted as listwise deletion in all relevant statistical tests.

## Supplementary information

Supplementary information.
