# Supplementary material for: Neurophysiological and behavioural markers of compassion
Source: Sci Rep. 2020 Apr 22;10:6789. doi: 10.1038/s41598-020-63846-3 (PMC7176659; doi:10.1038/s41598-020-63846-3)
Supplement: Supplementary file 1 — Supplementary information. [file 41598_2020_63846_MOESM1_ESM.docx]

Supplementary Information for

**Neurophysiological and behavioural markers of compassion**

Jeffrey J. Kim, Stacey L. Parker, James R. Doty, Ross Cunnington, Paul Gilbert, James N. Kirby

**Corresponding author:** Jeffrey J Kim

**Email:** Jeffrey.kim@uqconnect.edu.au

**This PDF file includes:**

Supplementary text

Figure S1

Table S1

SI References

Supplementary Information Text

**Procedure:** This project tracked 40 healthy participants across time, where first, with fMRI, we probed 40 healthy participants’ neural responses when engaged in either self-criticism verses self-reassurance. Second, a week later, we examined the same participants’ physiological correlates of engagement in a compassion meditation via measurement of heart-rate variability. Third, over a period of two-weeks, the same participants engaged in self-directed use of the compassion meditation via provision of the compassion meditation embedded as an audio link in a study website. Fourth, the same participants’ heart-rate variability was assessed when listening to the compassion meditation after the two-week training period.

**Fears of compassion scale:** We utilized the fears of compassion scale within the present research^1^, which has three subscales; measuring fear of compassion for the self (compassion we have for ourselves when we make mistakes or things go wrong in our lives), fear of compassion received from others (the compassion that we experience from others and flowing into the self), and fear of compassion for others (the compassion we feel for others, related to our sensitivity to other people’s thoughts and feelings).

**fMRI Stimuli Pretesting:** We assessed metrics of phrase length and phrase frequency to assess whether these might form potential confounds in the fMRI experiment. Phrase length compared the length of total character strings for each condition, which did not differ between the 30 emotional and 30 neutral statements (*p* = .662). Phrase frequency was assessed as clusters of words from the American Corpus of Contemporary English, to compare our stimuli to bins of low, medium, and high availability of words currently in use by text, speech, and academic writing. Three independent samples t-tests were conducted to represent each stimuli pair (emotional vs neutral) by the 3 bins (low, medium, high availability); none reached significance (*p* = .272, .773 and .404, respectively). Taken together, these findings indicate our stimuli are suitable for use in our fMRI Experiment.

Fig. S1. fMRI Task diagram for Experiment.

**
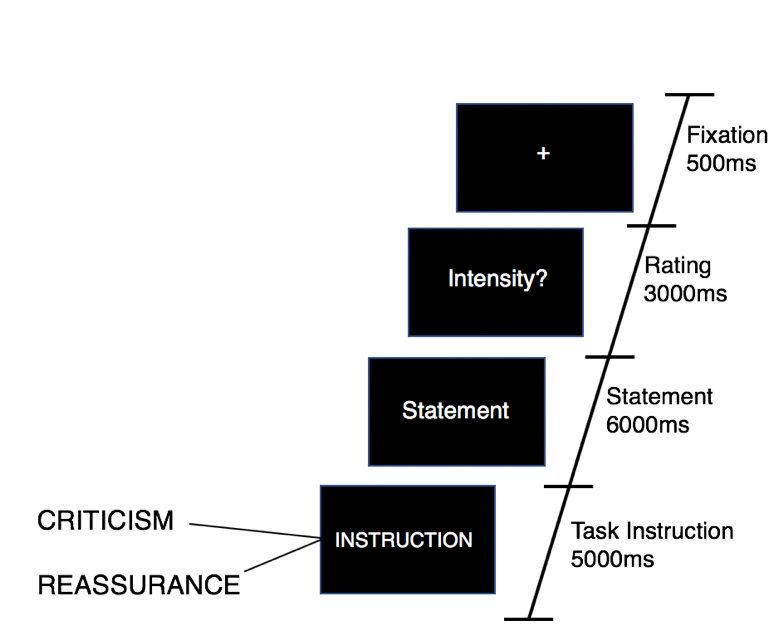
**

fMRI Task Diagram for Experiment probing Self-Criticism and Self-Reassurance. fMRI matlab code with corresponding stimuli can be found in the OSF link in the main manuscript.

**Table S1.** Brain regions that showed significantly greater activation for emotional versus neutral stimuli.

| Area | Cluster peak  co-ordinates | Peak  z-score | Cluster size | Cluster-level P_FWE_ |
| --- | --- | --- | --- | --- |
| *Emotional - Neutral* | | | | |
| Left Posterior Cingulate | -4 -52 36 | 5.82 | 135 | .0001 |
| Left Calcarine Gyrus | -10 -80 4 | 5.63 | 50 | .0001 |
| Right Lingual Gyrus | 14 -76 -10 | 5.54 | 49 | .0001 |
| Left Lingual Gyrus | 0 -68 6 | 5.46 | 46 | .0001 |
| Left Medial Frontal Gyrus | -2 46 36 | 5.39 | 25 | .0001 |
| Left Mid Cingulate | 0 -20 40 | 5.38 | 17 | .001 |

**References**

1. Gilbert P, Mcewan K, Matos M, Rivis A. Fears of compassion: Development of three self-report measures. 2011:239-255. doi:10.1348/147608310X526511
